# Effects of two immunosuppression regimens on T-lymphocyte subsets in elderly kidney transplant recipients

**DOI:** 10.3389/fimmu.2024.1405855

**Published:** 2024-09-20

**Authors:** Geraldo Rubens R. Freitas, Maria da Luz Fernandes, Fabiana Agena, Francine B. C. Lemos, Flavio J. de Paula, Verônica Coelho, Elias David-Neto, Nelson Z. Galante

**Affiliations:** ^1^ Serviço de Transplante Renal, Hospital das Clínicas, Faculdade de Medicina, Universidade de São Paulo, São Paulo, Brazil; ^2^ Departamento de transplante renal, Hospital Universitário de Brasília (HUB), Empresa Brasileira de Serviços Hospitalares (EBSERH), Brasília, Brazil; ^3^ Laboratório de Imunologia, Instituto do Coração, Hospital das Clínicas HCFMUSP, Faculdade de Medicina, Universidade de São Paulo, São Paulo, Brazil; ^4^ Laboratório de Investigação Médica 19 (LIM-19), Hospital das Clínicas HCFMUSP, Faculdade de Medicina, Universidade de São Paulo, São Paulo, Brazil; ^5^ Instituto de Investigação em Imunologia, Instituto Nacional de Ciência e Tecnologia (iii-INCT), São Paulo, Brazil

**Keywords:** regulatory (Treg) cell, kidney tranplantation, memory T CD4^+^ cells, elderly, everolimus

## Abstract

**Background:**

Despite the growing number of elderly kidney transplant (Ktx) recipients, few studies have examined the effects of immunosuppression on their lymphocyte profiles.

**Methods:**

We evaluated the early conversion from mycophenolate sodium (MPS) to everolimus (EVL) after rabbit antithymocyte globulin (rATG) 2 mg/kg induction in elderly kidney recipients. Three groups of KTx patients were compared: (a) Young (n=20, 36 ± 7 y) receiving standard immunosuppression (Group A1) (prednisone, tacrolimus, and MPS), (b) Elderly (n=35, 65 ± 3 y) receiving standard immunosuppression (Group B1), and (c) Elderly (n=16, 65 ± 3 y) with early (mean 30 d) conversion from MPS to EVL (Group B2). Naive, memory, and regulatory peripheral blood TCD4^+^ lymphocytes were quantified at 0, 30, and 365 d.

**Results:**

Results are reported as [mean(p25–p75)]. Young recipients had higher lymphocyte counts at baseline [2,100(1,630–2,400) vs. 1,310 (1,000–1,600)/mm^3^, p<0.0001] maintained higher counts within 365 d [1,850(1,590–2,120) vs. 1,130(460–1,325)/mm^3^, p=0.018 and vs. 1,410(805–1,895)/mm^3^, p=0.268]. Elderly recipients showed a decrease in lymphocytes within 30 d [1,310(1,000–1,600) vs. 910(700–1,198)/mm^3^, p=0.0012] with recovery within 365 d. The same pattern was observed in total lymphocytes and TCD4^+^ counts. Rabbit antithymocyte globulin induced a reduction in central memory T-cell percentages at 30 d in both young recipients [6.2(3.77–10.8) vs. 5.32(2.49–7.28)% of CD4^+^, p=0.036] and in elderly recipients [8.17(5.28–12.88) vs. 6.74(4.36–11)% of CD4^+^, p=0.05] on standard immunosuppression, returning to baseline at 365 d in elderly recipients but not in young recipients. Regulatory T CD39^+^ cells (Treg) percentages decreased at 30 d in elderly recipients [2.1(1.23–3.51) vs. 1.69(0.8–2.66)% of CD4^+^, p=0.0028] and in young recipients [1.29(0.45–1.85) vs. 0.84(0.18–1.82)% of CD4^+^, p=0.0038], returning to baseline at 365 d in elderly recipients [2.1(1.23–3.51) vs. 2.042(0.88–2.42)% of CD4^+^], but not in young recipients [1.29(0.45–1.85) vs. 0.86(0.7–1.34) % of CD4^+^]. The elderly everolimus conversion group did not show significant changes in cell profile over time or compared to elderly recipients with standard immunosuppression.

**Conclusion:**

Aging favored the maintenance of Treg during the late transplantation period despite ongoing immunosuppression. Lymphocyte depletion due to rATG was more prominent in elderly recipients and affected memory subsets with a temporary reduction in central memory T cells. However, conversion to everolimus did not impact Treg profile. Reducing the dose of rATG in elderly recipients seems necessary for the expected lymphocyte changes with EVL to occur.

**Clinical trial registration:**

nEverOld Trial, identifier NTC01631058.

## Introduction

A current challenge in kidney transplantation is to improve the short- and long-term outcomes for elderly recipients. The rates of infection (1) and malignancy (2) after transplantation are significantly higher among elderly recipients, while acute rejection episodes are less frequently reported (3). Renal-transplanted elderly patients exhibit higher rates of death-censored graft loss than non-elderly recipients (4), although their mortality is reduced when compared to those on dialysis (5).

Despite these clinical differences, and the likelihood of excessive immunosuppression (IS) in the elderly, it has been the usual practice to prescribe uniform IS regimens for all patients regardless of their age. Most clinical trials evaluating the efficacy and safety of new immunosuppression protocols focus on kidney transplant recipients in their fourth decade of life (ranging from 42.6 years to 49.3 years) (6–9). Our recent research showed that elderly recipients have reduced clearance of tacrolimus (TAC) (10) and a stable level of everolimus (EVL) despite lower oral doses (11), suggesting decreased metabolism of these drugs in the elderly patients. The divergent clinical outcomes observed between elderly and non-elderly transplant recipients highlight the need for a better understanding and tailored immunosuppression regiments for elderly recipients.

Recent guidelines recommend using biologic agents for induction therapy, suggesting interleukin-2 receptor-specific antibodies (IL2RA) for patients at low immunological risk, while rabbit anti-thymocyte globulin (rATG) is typically reserved for those at higher risk (12). However, despite these recommendations, there has been an increased use of rATG over IL2RA in both elderly and non-elderly recipients (13). This trend is driven by robust studies and meta-analyses indicating that rATG is associated with lower 5-year mortality (14) and acute rejection rates (15), with no differences in overall mortality (16) or allograft survival (15).

Among elderly recipients of low immunological risk, rATG is associated with lower acute rejection rates compared to IL2RA, while long-term outcomes remain similar (13). Despite the widespread use of rATG for induction therapy, its optimal dose, particularly for elderly patients, is not well established. Most transplant centers use a dose of 6 mg/kg, but lower doses may limit the duration of T-cell depletion without significantly affecting efficacy (17, 18). In elderly kidney transplant recipients receiving induction therapy with rATG 6 mg/kg, the rates of 1-year biopsy-proven acute rejection, infection and malignancy, and 3- year death-censored graft survival rates are comparable to those observed in non-elderly recipients (19).

Maintenance immunosuppression with mTOR inhibitors (mTORi) is emerging as a promising option for elderly transplant recipients. The anti-proliferative effects of mTORi is related to lower neoplasms incidence (20) and regression of myocardial hypertrophy (21). These benefits are particularly relevant for elderly recipients, who have higher rates of malignancies (2) and cardiovascular events (22). Recent trials have shown that EVL combined with a low dose of calcineurin inhibitor (CNI) is equivalent to the standard mycophenolate and CNI administration, in low immunological risk patients receiving rATG or basiliximab induction therapy (6). Furthermore, clinical trials with *de novo* EVL and reduced CNI exposure showed a significant lower incidence of cytomegalovirus (CMV) infection or disease compared to standard immunosuppression, even in the context of rATG induction and without pharmacological CMV prophylaxis (23, 24).

The lymphocyte profile in peripheral blood is important for predicting allograft outcomes. A higher frequency of peripheral blood regulatory T cells (CD4^+^CD25^+^FoxP3^+^) has been linked to stable graft function and immune tolerance (25), with lower acute rejection incidence (26), and positively correlated with higher glomerular filtration rates (26). Similarly, high numbers of regulatory B cells (CD19^+^CD24^Hi^CD38^Hi^) have been associated with stable graft function (27) and operational tolerance (28).

Immunosuppression directly affects both the quantitative and functional lymphocyte profiles of kidney transplant recipients. Induction therapy with rATG (29, 30) and maintenance immunosuppression with mTORi (31–33) tend to promote an immunomodulatory shift. In contrast, IL2RA and CNIs can lead to more profound immunosuppression, adversely impacting the survival and function of immunoregulatory cells (34). The influence of immunosuppression on the peripheral lymphocyte profile in elderly kidney transplant recipients has not been systematically evaluated.

The best immunosuppression for elderly kidney transplant recipients has not yet been precisely defined (35). Based on on previous observations, we hypothesized that using a lower-dose rATG induction and mTORi-based maintenance immunosuppression might better support immunoregulatory lymphocyte populations typically seen in the elderly. This approach could potentially reduce overall immunosuppression, avoiding occurrence of infections and malignancies associated with more intense immunosuppressive regimens. Our group conduced a prospective randomized clinical trial evaluating rATG induction with low-dose TAC and mTORi maintenance in the elderly population (36) (Clinical Trials Identifier: NTC01631058).

In this study, we evaluate rATG and mTORi effects on T-lymphocyte populations in elderly kidney transplant recipients.

## Materials and methods

### Study design and population

This study is a sub-analysis of patients included in the nEverOld trial (36) (Clinical Trials Identifier: NTC01631058) focusing on the characterization of peripheral lymphocyte profiles.

The nEverOld trial is an open-label, single-center, prospective, randomized, controlled trial designed to evaluate the efficacy and safety profile of low-dose rATG induction and early conversion to EVL in elderly kidney transplantation recipients compared to a standard of tacrolimus (TAC) and mycophenolate sodium (MPS).

We investigated the quantitative effects of rATG induction and EVL conversion on peripheral blood T-lymphocyte subpopulations by comparing three groups: (a) young kidney transplantation recipients with standard immunosuppression (Group A1) (n=20), (b) elderly kidney transplantation recipients with standard immunosuppression (Group B1) (n=19), and (c) elderly kidney transplantation recipients undergoing MPS to EVL conversion (Group B2) (n=16) along with low TAC.

Patients from June 2012 to December 2017 were included. All elderly patients (≥60 years) and undergoing their first kidney transplant, from deceased or living donors, were invited to participate in the study. Concurrently, a control group of younger patients, who received their kidney transplants consecutively after the elderly patients, was included for comparison.

According to the nEverOld trial design, elderly recipients were randomized at enrollment in a 1:1 ratio to either EVL conversion (Group B2) or standard immunosuppression (Group B1). For comparison, the elderly recipients in the EVL conversion group (Group B2) were also analyzed as if they were in the standard immunosuppression group (Group B1) before conversion.

Patients were excluded if their panel reactivity antibody was higher than 30%. All patients received induction therapy with methylprednisolone 500 mg and a single dose of rATG of 2.0 mg/kg. During the first month, all patients were given initial maintenance immunosuppression consisting of prednisone 0.5 mg/kg/d, tapered to 5 mg/d by the end of the first month, TAC 0.2 mg/kg/d b.i.d. adjusted to achieve a blood trough level between 8 ng/mL and 12 ng/mL, and a fixed dose of MPS at 720 mg b.i.d. After 30 d post-transplantation, young and elderly standard recipients (Group A1 and Group B1) continued with prednisone 5 mg/d, MPS 720 mg b.i.d., and TAC adjusted to maintain a trough blood level of 5–8 ng/mL. Patients in the elderly everolimus conversion group (Group B2) had EVL added to the MPS/TAC regimen at a dose of 1 mg b.i.d., with MPS reduced to 360 mg b.i.d. and TAC dose adjusted to achieve a blood trough level of 2–4 ng/mL. After 7 d, MPS was completely withdrawn, and EVL blood trough level was set to 3–8 ng/mL.

The study was approved by institutional board of ethics in research (CAPPesq no. 44943215.0.0000.0068). All individuals provided informed consent prior to enrollment.

### Times for blood sample collection and cell preparation

Blood samples were collected from all groups at day 0, 30, and 365 after transplantation. The elderly everolimus conversion group (Group B2) also collected an additional sample 30 d after EVL conversion, referred to as day 60. Since the elderly groups were under the same immunosuppression regimen from baseline up to 30 d post-transplantation, they were analyzed together for these time points. At day 60 and day 365 post-transplantation, samples were analyzed according to their respective groups.

Peripheral blood mononuclear cells (PBMCs) were separated using Ficoll density gradient centrifugation and cryopreserved for further analysis.

In the original study design, we planned to analyze the effect of everolimus on elderly patients 30 d after conversion. Samples were collected at 0 d, 30 d, and 60 d, and the PBMCs were cryopreserved for subsequent analysis. However, an interim analysis of data from 50% of the included patients showed that the anticipated differences with everolimus use in the elderly could be more evident in the late post-transplant. As a result, we decided to extend the sample collection period to up to 1 year post-transplant. Unfortunately, few patients were available for this extended collection period, leading to a limited number of samples at 365 d.

### Flow cytometry identification of T- lymphocyte sub-populations

PBMCs were stained with titrated mouse anti-human monoclonal antibodies. Anti-CD4-fluorescein isothiocyanate (FITC) (OKT4), anti-CCR7 (CD197)-Phycoerythrin (PE) (3D12), anti-CD45RA-PE-Cy7 (HI100), anti-CD25-PE (BC96), anti-CD127-PE-Cy7 (RDR5), anti-FoxP3-Peridinin Chlorophyll Protein Complex (PerCp)-Cy5.5 (PCH101), and anti-CD39- allophycocyanin (APC) (A1) antibodies were from eBiosciences (San Diego, CA, USA). Anti-CD3-APC-Cy7 (SK7), anti-CD8-AmCyan (SK1), and BD Multitest™ CD3/CD8/CD45/CD4 [anti-CD3-FITC (SK7), anti-CD8-PE (SK1), anti-CD45-PerCP (2D1 (HLe-1), and anti-CD4-APC (SK3)] were from BD Biosciences (Heidelberg, Germany). For intra-cellular staining of FoxP3, cells were washed, fixed, and permeabilized with FoxP3 staining buffer from eBioscience (San Diego, CA, USA) immediately after surface staining. At least 0.5×10^5^ events in the lymphocyte region were acquired. Fluorescence minus one (FMO) controls were set up for CD127, FoxP3, CD39, CCR7 (CD197), and CD45RA markers.

**Figure 1:**
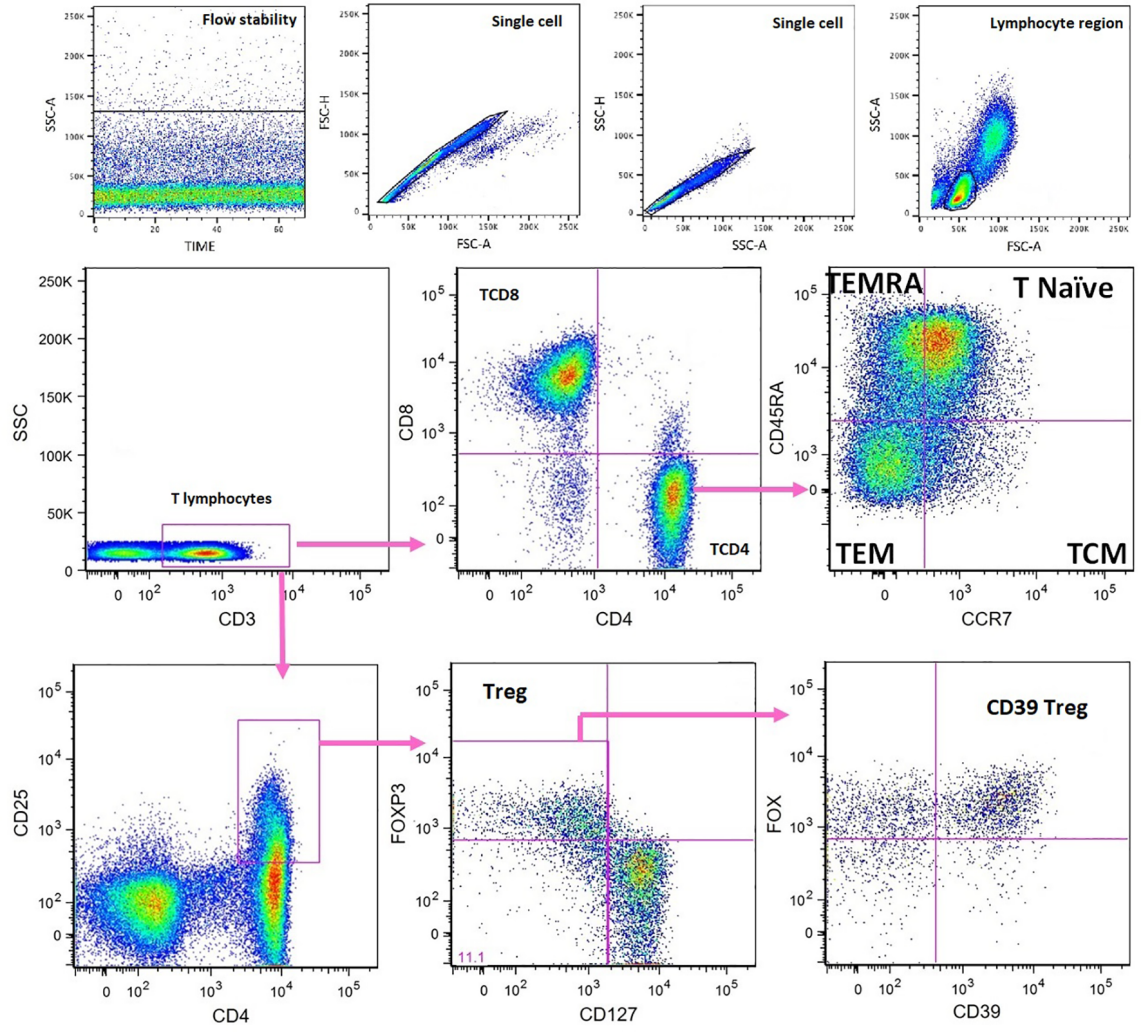
Flow cytometry characterization of peripheral blood T- cell subsets. Fluorescence minus one (FMO) controls were set up for CD127, FoxP3, CD39, CCR7 (CD197), and CD45RA. TCM, T central memory; TEM, T effector memory; TEMRA, terminally differentiated effector memory; Treg, regulatory T cells.

Flow cytometry was performed in a FACSCanto-II (BD Biosciences) cytometer. FlowJo 9.1 software (TreeStarInc, San Carlos, CA, USA) was used for analysis. After exclusion of cell doublets and debris, sequential gating of PBMC was performed in the lymphocyte region. The gating strategies used to define T-cell subsets are shown in **Figure 1**. The lymphocyte subsets evaluated were T (CD45^+^CD3^+^), TCD8 (CD45^+^CD3^+^CD8^+^), and TCD4 (CD45^+^CD3^+^CD4^+^), and the subpopulations TCD4 naive (CD3^+^CD4^+^CCR7^+^CD45RA^+^) (T naive), TCD4 central memory (TCM) (CD3^+^CD4^+^CCR7^+^CD45RA^−^), TCD4 effector memory (TEM) (CD3^+^CD4^+^CCR7^−^CD45RA^−^), TCD4 terminally differentiated effector memory (TEMRA) (CD3^+^CD4^+^CCR7^−^CD45RA^+^), regulatory TCD4 (Treg) (CD3^+^CD4^+^CD25^hi^CD127^−^FoxP3^+^), and regulatory TCD4^+^CD39^+^ (CD39Treg).

Absolute counts of lymphocyte subsets were calculated using percentages obtained from flow cytometry and lymphocyte counts from standard blood counts performed on fresh blood before PBMC separation. The subset percentages analyzed were referred to as total lymphocyte counts for T, TCD4, TCD8, to TCD4 cells for T naive, TCM, TEM, and TEMRA and to TCD4CD25^hi^ cells for Treg and CD39^+^Treg.

### Statistical analyses

The Kolmogorov–Smirnov or Shapiro–Wilk tests were used to assess the normality of continuous variables. For comparisons of normally distributed continuous variables, Student’s t-test was used, while the Mann–Whitney U-test was applied for non-normally distributed variables. In paired sample analyses, the paired Student’s t-test was employed for normally distributed variables, and the Wilcoxon test was used for non-normally distributed variables. Nominal variables were compared using the chi-square or Fisher’s exact test. Data are presented as medians and interquartile ranges [median (p25–p75)]. A p-value ≤0.05 was considered statistically significant.

All analyses were performed with SPSS-20 (IBM-Corp., Armonk, NY, USA) and GraphPad Prism 6 software (GraphPad Software Inc., La Jolla, CA, USA).

## Results

**Table 1** presents the demographics and transplant characteristics of the different groups. **Table 2** provides details on TAC and EVL trough blood levels.

**Table 1:** Recipient demographics according to studied groups.

Characteristics	Group A1 (n=20)	Group B1 (n=19)	Group B2 (n=16)
Age (years)	36 ± 7	65 ± 3^a^	65 ± 3^b^
Male gender n(%)	8 (40)	10 (52.6)	10 (62.5)
Comorbidity n(%)			
SAH	18 (90)	19 (100)	16 (100)
DM	4 (20)	11 (57.9)	6 (37.5)
Underlying renal disease n(%)			
Diabetic nephropathy	3 (15)	10 (52.6)	4 (25)
Vascular nephropathy	1 (5)	5 (26.3)	4 (25)
Glomerulonephritis	3 (15)	1 (5.3)	1 (6.2)
ADPKD	1 (5)	1 (5.3)	1 (6.2)
Others	12 (60)	2 (10.5)	6 (37.5)
HD n(%)	19 (95)	16 (84.2)	16 (100)
Time of RRT (months)	30.5 (14–39.25)	38 (21–54)	33.5(20.75–49.75)
PRA	0 (0–0)	26 (10.5–43)	18 (5.75–39.25)
HLA-mismatches	4 (3–4.75)	2 (2–4)	3 (2–3.75)
Deceased donor n(%)	9 (45)	19 (100)^a^	14 (87.5)^b^
Expanded criteria donor n(%)	2 (22.2)	11 (57.9)	6 (42.9)
Cold ischemia time (hours)	24 (22–33.5)	26 (21–28)	24 (21.5–27.5)

Group A1, young standard immunosuppression; Group B1, elderly standard immunosuppression; Group B2, elderly everolimus conversion; n, number; SAH, systemic arterial hypertension; DM, diabetes mellitus; ADPKD, autosomal dominant polycystic kidney disease HD, hemodialysis; RRT, renal replacement therapy, PRA, panel reactivity antibody; HLA, human leukocyte antigen.

^a,b^p<0.05 for Group A1 vs. Group B1 and Group A1 vs. EC groups, respectively.

**Table 2:** Number of available samples and immunosuppressant drugs blood levels according to time after transplantation.

	0d	30d	60d	365d	TAC30d	TAC60d	TAC365d	EVL60d	EVL365d
Group A1	20	17	0	7	11.2 ± 2.7	–	6.5 ± 2	–	–
Group B1 analysis	34	33	0	5	–	–	–	–	–
Group B1	19	18	0	5	10.7 ± 4.3	–	7.4 ± 2.1	–	–
Group B2	15*	15*	14	5	9.4 ± 2.1	4.7 ± 1.6	3.3 ± 1.5	5.8 ± 4	5.4 ± 2.2

Group A1, young standard immunosuppression; Group B1, elderly standard immunosuppression; Group B2, elderly everolimus conversion; d, days; TAC, tacrolimus; EVL, everolimus. Group B1 analysis refers to all elderly patients before conversion.

*Patients of Group B2 group included in Group B1 in the initial analysis. TAC and EVL blood levels are expressed in ng/mL.

In the young control group (Group A1), the median daily dose of mycophenolate sodium was 1,440 mg at 30 d and 90 d and 1,080 mg at 365 d. In the elderly group with standard immunosuppression (Group B1), the median daily dose was 1,440 mg at both 30 and 90 d and 900 mg at 365 d. Consequently, the proportion of patients deviating from the protocol-specified dose at 30, 90, and 365 d was 10%, 25%, and 71% in Group A1, and 21%, 38.8%, and 64.3% in Group B1, respectively.

Recipients in the young standard immunosuppression group (Group A1) were approximately 30 years younger than those in both the elderly groups, standard immunosuppression (Group B1) (36 ± 7 y vs. 65 ± 3y, p < 0.05) and everolimus conversion (Group B2) (36 ± 7 y vs. 65 ± 3 y, p<0.05). The mean ages of the elderly recipients in groups B1 and B2 were similar. There were no differences between the groups in terms of gender, rates of systemic arterial hypertension (SAH) and diabetes mellitus (DM), time on dialysis, number of human leukocyte antigen (HLA)-A, HLA-B, and HLA-DR mismatches, percentages of panel reactivity antibodies, expanded criteria donors, and cold ischemia time. However, elderly recipients received grafts from deceased donors more frequently than their younger counterparts (100% vs. 45%, p<0.05 for B1; 87.5% vs. 45%, p<0.005 for B2) (**Table 2**).

### Quantification of total lymphocyte and T subsets

**Tables 3**, **4** show absolute lymphocyte counts over times.

**Table 3:** Absolute numbers (cells/mm3) of lymphocyte subpopulations according to studied groups and observation time.

Lymphocyte subset	Group A1			Group B1			Group B2	
	Pre-transplantation	Day 30	Day 365	Pre-transplantation	Day 30	Day 365	Day 60	Day 365
Total Lymphocytes	2,100 (1,630–2,400)	1,960 (1,270–2,970)	1,850 (1,590–2,120)	1,310 (1,000–1,600)^a^	910 (700–1,198)^b,^*	1,130 (460-1,325)^c^	1,030 (800–1,230)	1,410 (805–1,895)
T	1,334 (848.4–1,633)	1,202 (707–1,619)	1,333 (941.7–1,507)	695.5 (509.8–952)^a^	471.9 (182.4–754.7)^b,^*	344.7 (293–884.2)^c^	599.5 (271.3–802.6)	799.5 (539.4–1,177)
TCD8	391.1 (298.4–494.9)	443.3 (251.8–747.3)	426.5 (376.8–731.9)	206.3 (145.7–332.1)^a^	160.2 (82.55–246.3)^b^	145.4 (122.8–369)^c^	173.9 (106–320.6)	363.8 (211.7–461.7)
TCD4	748.7 (588.6–983.4)	706.5 (340.8–833.3)	743.1 (453.9–958.2)	463.5 (277.6–631.2)^a^	245.3 (87.06–475.3)^b,^*	194 (143.3–473.2)^c^	345.2 (123.5–549.6)	400.5 (293.2–683.9)
T naive	116.9 (53.76–293.3)	189.6 (19.44–298.7)	204 (63.89–343)	71.47 (32.11–137.9)^a^	50.06 (14.87–130.9)^b^	37.89 (26.43–134.6)	75.19 (13.76–124.7)	132.1 (51.86–153.6)
TCM	61.01 (19.77–87.24)	26.18 (13.01–48.87)	57.1 (26.57–80.98)	36.02 (22.73–65.39)	17.7 (5.61–36.34)*	51.46 (27–87.59)	18.04 (3.28–27.01)	47.55 (24.36–107.7)
TEM	252.5 (172.9–353.4)	162.2 (89.91–214.4)*	274.3 (188.8–398.6)^‡^	181.4 (99.22–245.4)^a^	97.85 (39.91–156.6)^b,^*	155.5 (77.08–199.5)^c^	88.08 (51.26–161.5)	177.3 (145.8–356.1)
TEMRA	274.5 (82.54–322.3)	159.8 (73.19–386.2)	81.61 (62.88–212.6)	114.5 (63.2–179.2)^a^	83.7 (15.15–141.3)^b^	12.9 (12.39–42.57)^c^	103.3 (35.24–235.4)^†^	27.06 (21.97–130.8)
Treg	17.72 (13.49–33.5)	12.44 (7.31–19.62)	17.57 (8.91–30.73)	25.05 (10.41–30.17)	8.554 (4.41–15.23)*	9.46 (3.57–14.8)	9.13 (3.25–17.8)	14.41 (7.37–46.18)
CD39Treg	12.9 (3.24-18.06)	3.89 (1.81–8.59)*	5.83 (1.19–8.58)***	9.434 (4.99–18.06)	4.305 (1.99–8.01)*	3.27 (2.1–7.73)	2.2 (0.97–5.88)**	1.98 (1.35–5.48)

Group A1, young standard immunosuppression; Group B1, elderly standard immunosuppression; Group B2, elderly everolimus conversion; TCM, T central memory; TEM, T effector memory; TEMRA, terminally differentiated effector memory; Treg, regulatory T cells.

^a,b,c^ p<0.05 for Group A1 vs. Group B1 pre-transplantation, day 30 and day 365 samples, respectively.

*,**,*** p<0.05 for pre-transplantation vs. day 30, pre-transplantation vs. day 60 and pre-transplantation vs. day 365 samples of the same group, respectively.

^†,‡^ p< 0.05 for day 30 vs. day 60 and day 30 vs. day 365 samples of the same group, respectively.

**Table 4:** Percentages of lymphocyte subpopulations according to studied groups and observation time.

Lymphocyte subset	Group A1			Group B1			Group B2	
	Pre-transplantation	Day 30	Day 365	Pre-transplantation	Day 30	Day 365	Day 60	Day 365
T	69.95 (61.7–77.4)	72.1 (44.15–75.95)	71.1 (58.8–79.8)	59.35 (42.8–70.5)^a^	58.9 (30.6–71.85)	66.1 (44.75–68.8)	62.55 (44.8–68.8)	59.8 (55.65–71.35)
TCD8	20.23 (17.07–29)	22.29 (17.01–34.63)	26.29 (23.46–33.1)***	17.6 (13.46–23.61)	18.3 (10.49–23.3)^b^	27 (17.02–32.92)	17.52 (11.03–26.01)	26.31 (21.02–31)
TCD4	36.49 (31.46–45.5)	26.84 (20.43–37.06)	42.49 (22.3–42.95)	32.62 (24.9–41.2)	29.44 (15.3–40.33)	30.04 (20.81–32.42)	33.55 (17.83–43.17)	25.82 (24.92–39.18)
T naive	21.4 (7.72–36.85)	27.2 (16.6–41.7)	27.6 (10.6–41.7)	18.55 (6.9–32.83)	23.1 (12.15–30.8)	22.1 (13.35–31.65)	25.2 (12.08–32.98)	22.5 (15.5–32.6)
TCM	6.2 (3.77–10.8)	5.32 (2.49–7.28)*	8.62 (4.95–13.5)	8.17 (5.28–12.88)	6.74 (4.36–11)*	21.2 (10.94–27.95)^c^	5.47 (3.14–7.95)**	11.1 (8.05–18)
TEM	34.05 (24.53–51.7)	25 (21.35–42.05)	44.2 (28.5–63)	41.55 (29.95–51.33)	33.5 (28.85–44.5)^b^	50.6 (44.55–54)	30.3 (26.55–48.83)	50.1 (45.3–57.1)
TEMRA	32.8 (21.43–43.38)	36.2 (22–44.5)	15.7 (9.05–30.2)	24.85 (21.13–37.48)	28.3 (22.5–41.55)	7.22 (5.55–11.4)^c^	29.65 (25.45–44)	13 (6.02–19.15)
Treg	2.2 (1.8–3.79	1.82 (1.5–2.28)*	2.15 (2.09–4.28)	3.99 (2.62–5.54)^a^	2.96 (2.37–4.4)^b,^*	3.05 (2.68–3.38)	3.14 (1.81–5.26)	4.25 (3.27–8.58)
CD39Treg	1.29 (0.45–1.85)	0.84 (0.18–1.82)*	0.86 (0.70–1.34)***	2.1 (1.23–3.51)^a^	1.69 (0.8–2.66)^b,^*	2.042 (0.88–2.42)	1.02 (0.56–1.74)**	0.83 (0.41–1.09)

Group A1, young standard immunosuppression; Group B1, elderly standard immunosuppression; Group B2, elderly everolimus conversion; TCM, T central memory; TEM, T effector memory; TEMRA, Terminally differentiated effector memory; Treg, regulatory T cells.

^a,b,c^ p<0.05 for Group A1 vs. Group B1 pre-transplantation, day 30 and day 365 samples, respectively.

*,**,*** p<0.05 for pre-transplantation vs. day 30, pre-transplantation vs. day 60 and pre-transplantation vs. day 365 samples of the same group, respectively.

**Figure 2:**
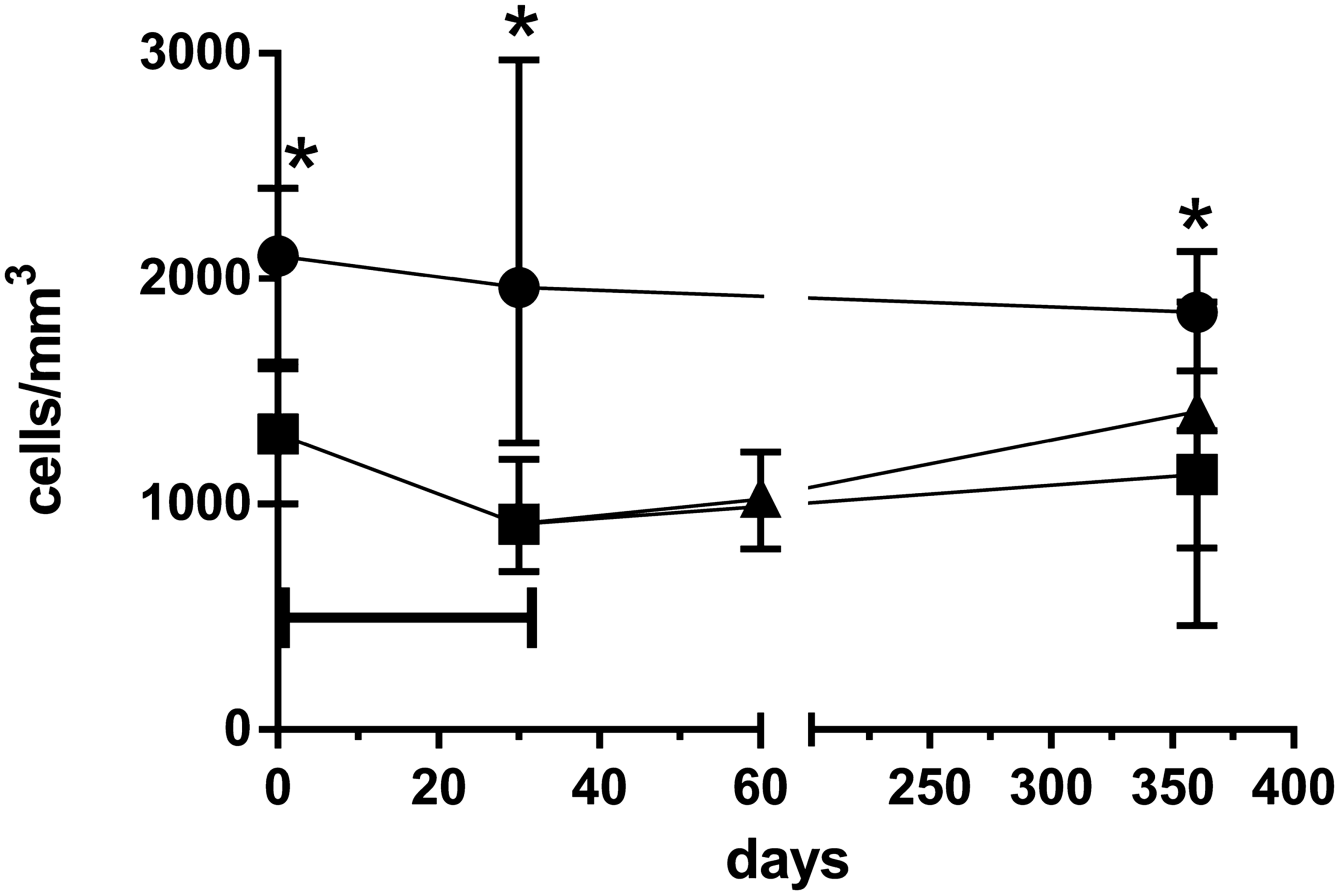
Total lymphocyte absolute counts over 365 days observation time for young standard immunosuppression (Group A1) (•), elderly standard immunosuppression (Group B1), (▪) and elderly everolimus conversion (Group B2) (▴) groups. *p<0.05 for comparison between Group A1 vs. Group B1 groups in a given time point. Horizontal bar p<0.05 for comparison between day 30 and pre-transplantation samples of Group B1.

Recipients in the young standard immunosuppression group (Group A1) had higher total lymphocyte absolute counts than elderly on standard immunosuppression (Group B1) at days 0, 30, and 365. Following induction therapy with a single rATG dose (2 mg/kg) elderly recipients in Group B1 experienced a reduction in total lymphocyte counts at 30 d (p=0.0012), but counts returned to pre-transplantation levels by the end of the first year. In the elderly everolimus conversion group (Group B2), total lymphocyte counts recovered to pre-transplantation values 30 d after conversion to EVL and remained stable at 365 d, showing no significant difference compared to the young standard immunosuppression group (Group A1) (**Table 3**; **Figure 2**). Throughout the study period, the young standard immunosuppression group (Group A1) consistently exhibited higher absolute counts of total T, TCD4, and TCD8 lymphocytes compared to the elderly standard immunosuppression group (Group B1). However, the elderly everolimus conversion group (Group B2) showed an increase in total T lymphocyte counts, mainly due to an increase in TCD8 counts (p=0.0625), at 365 days (**Table 3**; **Supplementary Figure S1**).

### Quantification of TCD4 lymphocyte subsets

At baseline, we observed only a few differences between the young and elderly groups on standard immunosuppression (Group A1 and Group B1). The young group had higher percentages of T-naive cells (p=0.0619) (**Table 4**). However, the absolute numbers of T-lymphocyte subsets (TCD4, TCD8, Tnaive, TEM, and TEMRA) were lower in the elderly group (p = 0.017, p = 0.001, p = 0.049, p = 0.033, p = 0.012, respectively) with the exception of central memory T cells (TCM) (p = 0.177) and Tregs (p = 0.712), which showed no significant differences (**Table 3**; **Supplementary Figure S1**).

**Figure 3:**
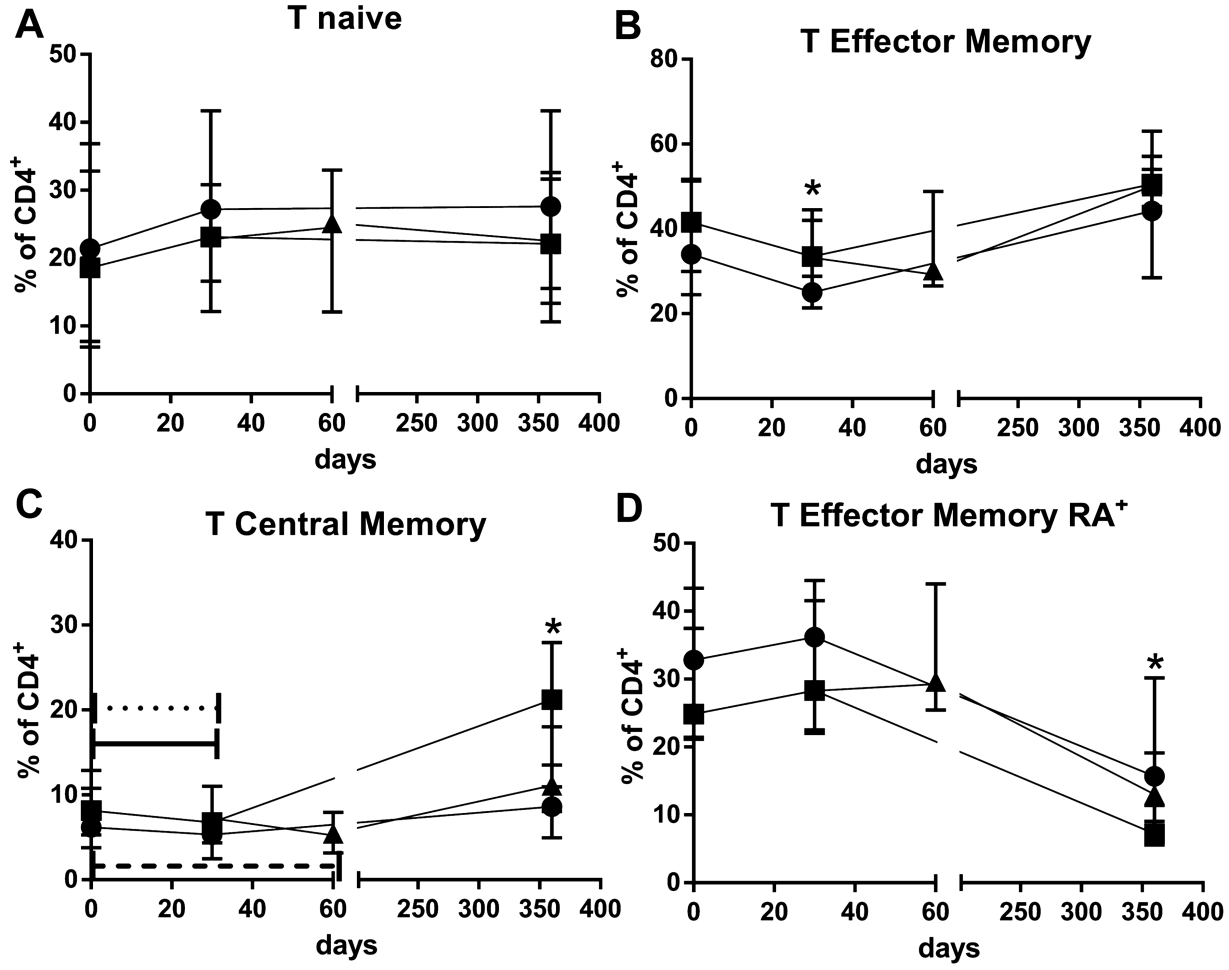
Percentages of T CD4^+^naïve **(A)**, T CD4^+^ effector memory **(B)**, T CD4^+^central memory **(C)** and T CD4^+^ TCD4 terminally differentiated effector memory **(D)** lymphocytes over 365 days observation time for young standard immunosuppression (Group A1) (•), elderly standard immunosuppression (Group B1) (▪) and elderly everolimus conversion (Group B2) (▴) groups. *p<0.05 for comparison between Group A1 vs. Group B1 groups in a given time point after transplantation. Dotted, solid, and dashed horizontal bars p<0.05 for comparisons between a given time point and the pre-transplantation samples of Group A1, Group B1, and Group B2, respectively.

At baseline, there was a trend towards higher percentages of TCM, TEM, and TEMRA in the elderly on standard immunosuppression (Group B1) and higher percentages of T-naive in the young on standard immunosuppression (Group A1) (**Table 4**; **Figure 3**).

Following induction therapy with rATG and immunosuppression, there was a reduction in the percentages of central memory T cells at 30 d post-transplantation (p=0.0498 and p=0.0359, respectively). By 365 d, the percentage of central memory T cells had returned to baseline values in the elderly with standard immunosuppression but not in the young. Thirty days after EVL conversion, there were no changes in the percentage of central memory T cells, and no differences were observed compared to elderly with standard immunosuppression (Group B1), at 365 d (**Figure 3C**; **Table 4**).

At 365 d, there was a trend towards a reduction in the percentages of terminally differentiated effector memory T cells across all groups. At 365 d, the young group (Group A1) exhibited a higher percentage of terminally differentiated effector memory T cells compared to the elderly group with standard immunosuppression (Group B1) (p = 0.048). EVL conversion did not affect the percentages of terminally differentiated effector memory T cells in the elderly groups (**Figure 3D**; **Table 4**).

Throughout the study period, we did not observe significant changes in the percentages of T-naive and effector memory T cells in any of the groups (**Figures 3A, B**). Interestingly, EVL initiation had an opposite effect on the percentages of effector memory and central memory T cells, inducing an increase in the percentage of effector memory T cells and a decrease in percentages of central memory T cells (**Figures 3B, C**; **Table 4**).

### Regulatory T cells

At baseline, elderly recipients (Group B1) had significant higher percentage of CD39Treg lymphocytes than young recipients (Group A1) (p = 0.015). Following induction with rATG, there was a significant decrease in the percentage of these cells in both groups (Group B1: p = 0.0028, Group A1: p = 0.0038). By 365 d, CD39Treg cells had recovered to the baseline values in the elderly with standard immunosuppression (Group B1) but not in young (Group A1) (p = 0.0156).

**Figure 4:**
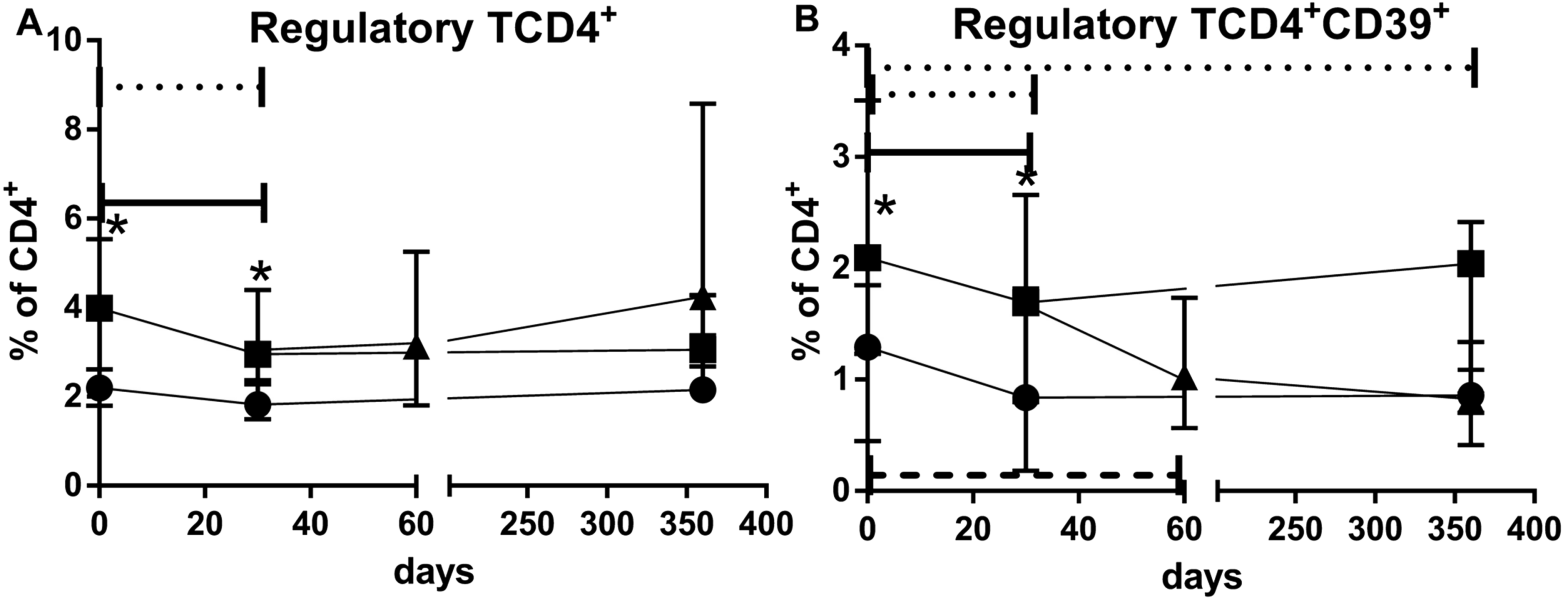
Percentages of regulatory **(A)** and CD39^+^regulatory **(B)** T cells over 365 days observation time for young standard immunosuppression (Group A1) (•), elderly standard immunosuppression (Group B1) (▪), and elderly everolimus conversion (Group B2) (▴) groups. *p<0.05 for comparison between Group A1 vs. Group B1 groups in a given time point after transplantation. Dotted, solid, and dashed horizontal bars p<0.05 for comparisons between a given time point and the baseline percentages of Group A1, Group B1, and Group B2, respectively.

However, conversion to EVL did not allow the recovery of CD39Treg to baseline levels by 365 d, in contrast to the elderly with standard immunosuppression (Group B1) (**Figure 4B**; **Table 4**). These differences were less pronounced when only Treg cells were analyzed (**Figure 4A**; **Table 4**).

### Clinical data

In the study, there were seven Banff borderline changes observed: four in Group A1 and three in Group B1. Among Group A1, there was one case of humoral rejection. Each group experienced one case of cellular rejection: Group A1, Group B1, and Group B2. Additionally, there were four cases of renal graft loss within the first year: one in the Group A1 control group and three in Group B2, with one graft loss due to death in the elderly group. Regarding infectious complications, BK virus infections were noted in three Group A1, three Group B1, and six Group B2 patients, while CMV infections occurred in three, two, and zero patients, respectively.

## Discussion

We analyzed the impact of two different immunosuppression regimens on T-lymphocyte subsets in elderly kidney transplant recipients and compared the results with a younger control group under a standard regimen.

Our data showed that a single very low dose (2 mg/kg single dose) of rATG induction therapy combined with standard immunosuppression in elderly recipients resulted in early reduction in the total T lymphocytes, which only recovered within 1 year of transplantation, regardless of MPS maintenance or everolimus conversion. Additionally, analysis of TCD4^+^ lymphocyte subsets in the elderly indicated significant early reductions in TCM and Treg cell population percentages, which also recovered after 1 year with TAC/MPS maintenance immunosuppression. These reduction effects on TCM and Treg cells were not observed in the young adult group or in the elderly everolimus conversion group. Both groups showed an early reduction in the percentages of TCM and Treg cells, which was sustained throughout the 1-year follow-up period.

Elderly recipients with standard immunosuppression consistently had lower total lymphocyte counts compared to the younger controls throughout the observation period. This finding aligns with our previous research, which identified this as an age-dependent effect, particularly pronounced among end-stage renal disease patients (37).

Early after transplantation with rATG induction at a single 2 mg/kg dose, the total lymphocyte counts significantly decreased among elderly recipients but not among the young controls. At the end of 1-year follow-up, both elderly and young recipients with standard immunosuppression had their total lymphocyte counts return to baseline. Although the intensity and duration of rATG-related lymphocyte depletion are dose dependent (38), several factors may contribute to the lower capacity for T-cell reconstitution in elderly recipients. These factors include a reduced rate of thymic output (39–41), lower proliferative capability of depletion-resistant cells (42), and a higher rate of apoptosis of T cells in elderly recipients.

Given that the rATG dose used in this study was much lower than a conventional dose, we suggest that even a low dose rATG induction combined with standard immunosuppression has a significant, yet transient, impact on T- lymphocyte counts in elderly individuals.

The T- lymphocyte subset analysis of elderly recipients with standard immunosuppression showed significant early reductions of TCD4 and TCM cell populations, which recovered 1 year after transplantation. These effects were anticipated, considering that antibodies against surface antigens of TCD4 lymphocytes predominate in rATG preparations (43), leading to significant changes in the composition of this T- lymphocyte subsets after rATG therapy (29, 44–46).

Contrarily, the observed changes in TCD4 cells subsets in this study do not align with the reported relative resistance of memory cells to rATG depletion (29, 45, 46). Additionally, the percentages and frequencies of memory cells are predictors of the risk of the kidney allograft failure (47). However, to the best of our knowledge, studies directly addressing possible differential effects of rATG or maintenance immunosuppression on lymphocyte subsets according to the age of the recipients are not available. Notably, most studies on the effects of rATG on TCD4 lymphocytes refers to a 6-mg/kg dose in both clinical (29, 44, 46) and *in vitro* observations (30).

Elderly recipients with standard immunosuppression showed significant early reductions of Treg cell populations. These reductions in absolute counts and percentages of CD39Treg cell population contrast with *in vitro* reports, indicating that rATG therapy favors Treg expansion (30). Similarly, *in vivo* data have shown preserved Treg cell functional activity even when associated with standard maintenance immunosuppression based on calcineurin inhibitors (29, 44, 46, 48). Although substantial evidence indicates that inhibition of mTOR signaling pathway with rapamycin effectively expands Tregs *in vitro* (49, 50), and that rapamycin, either in combination with mycophenolate and corticosteroids (32, 33), or as monotherapy (51), promotes the survival and expansion of cells with regulatory function in clinical settings, these effects were not observed in the elderly patients in this study.

In this study, early TAC levels were 11.2 ± 2.7 ng/mL in young controls and 10.7 ± 4.3 ng/mL in elderly recipients, with lower levels during follow-up, 6.5 ± 2 ng/mL and 74 ± 2.1 ng/mL, respectively. These levels were much higher than those in the everolimus conversion group, which had a TAC level of 3.3 ± 1.5 ng/mL. Since Treg generation, survival, and function are IL-2 dependent (52), we speculate that concomitant use of calcineurin inhibitor may account for the observed impairment in the expected Treg expansion. Additionally, the later reduction in target levels of tacrolimus resulted in an increase in Treg counts in elderly recipients.

Our study compares *de novo* standard tacrolimus-based immunosuppression with everolimus-based immunosuppression combined with low-dose tacrolimus. In the conversion group, the target TAC blood levels were maintained at 4 ng/mL throughout the study period. Both sirolimus and EVL can compete with TAC for the binding to the FKBP12 (53). Tacrolimus has been reported to interfere with the actions of mTOR inhibitors (mTORis), as seen in clinical settings such as BK virus replication. While sirolimus inhibits BKV proliferation *in vitro*, tacrolimus, at blood concentrations between 5 ng/mL and 10 ng/mL, reverses this effect (54).

Based on these observations, we hypothesize that maintaining tacrolimus levels in the everolimus-based immunosuppression regimen may have inhibited the beneficial effects of everolimus on Treg expansion. Additionally, the lack of benefit from mTOR inhibitors in promoting Treg expansion among elderly recipients might be related to immune senescence. Most studies assessing the effects of mTOR inhibitors on Treg immunobiology have involved younger individuals (32, 33, 51).

Our study is limited by a small sample size, which reduces statistical power. This limitation also affects the generalizability of our findings and can amplify random variations, making it challenging to distinguish true effects from noise. However, to the best of our knowledge, no previous studies have provided this information about immune cells in this specific group of kidney transplant recipients.

Given the limited number of clinical events, such as graft loss, rejections, and deaths, we were unable to evaluate the specific impact of lymphocyte subpopulations on clinical outcomes. Additionally, the cytometry gating strategy used did not include CD8 labeling, preventing analysis of TCD8^+^ lymphocyte populations. Although we evaluated a broad range of lymphocyte phenotypes, we did not conduct cell functional assays, which could have provided valuable insights, especially considering that elderly recipients generally have lower rates of immune activation and kidney transplant rejection.

In conclusion, low dose of rATG affected memory subsets in elderly recipients. Everolimus- based immunosuppression did not show a favorable effect in the Treg profile. Aging favored Treg maintenance during the later stages of transplantation, independent of the type of maintenance immunosuppression.

## Data Availability

The raw data supporting the conclusions of this article will be made available by the authors, without undue reservation.

## In memoriam

We acknowledge Dr. Flavio Jota de Paula for his lifework in our institution.

## Supplementary material

Supplementary Figure 1 T **(A)**, TCD4^+^ **(B)** and T CD8^+^ **(C)** lymphocyte absolute counts over 365 days observation time for young standard immunosuppression (GROUP A1) (•), elderly standard immunosuppression (GROUP B1) (▪) and elderly everolimus conversion (GROUP B2) (▴) groups. * p<0.05 for comparison between GROUP A1 vs. GROUP B1 groups in a given time point. Horizontal bar p<0.05 for comparisons between 30 day and baseline cell counts of GROUP B1 group.

## Glossary

APC: allophycocyanin
CD39Treg: regulatory TCD4+CD39+
CMV: cytomegalovirus
CNI: calcineurin inhibitor
d: days
DM: diabetes mellitus
Group B2: elderly everolimus conversion
Group B1: elderly standard immunosuppression
EVL: everolimus
FITC: fluorescein isothiocyanate
FMO: fluorescence minus one
HLA: human leukocyte antigen
IL2RA: interleukin-2 receptor specific antibodies
IS: immunosuppression
Ktx: kidney transplant
MPS: mycophenolate sodium
mTORi: mTOR inhibitors
PBMC: peripheral blood mononuclear cells
PE: phycoerythrin
PerCp: peridinin chlorophyll protein complex
rATG: rabbit antithymocyte globulin
SAH: systemic arterial hypertension
TAC: tacrolimus
TCM: TCD4 central memory
TEM: TCD4 effector memory
TEMRA: TCD4 terminally differentiated effector memory
Treg: regulatory TCD4
y: years
Group A1: Young standard immunosuppression group.
