# Supplementary figures and images for: Effects of two immunosuppression regimens on T-lymphocyte subsets in elderly kidney transplant recipients
Source: Front Immunol. 2024 Sep 20;15:1405855. doi: 10.3389/fimmu.2024.1405855 (PMC11449757; doi:10.3389/fimmu.2024.1405855)

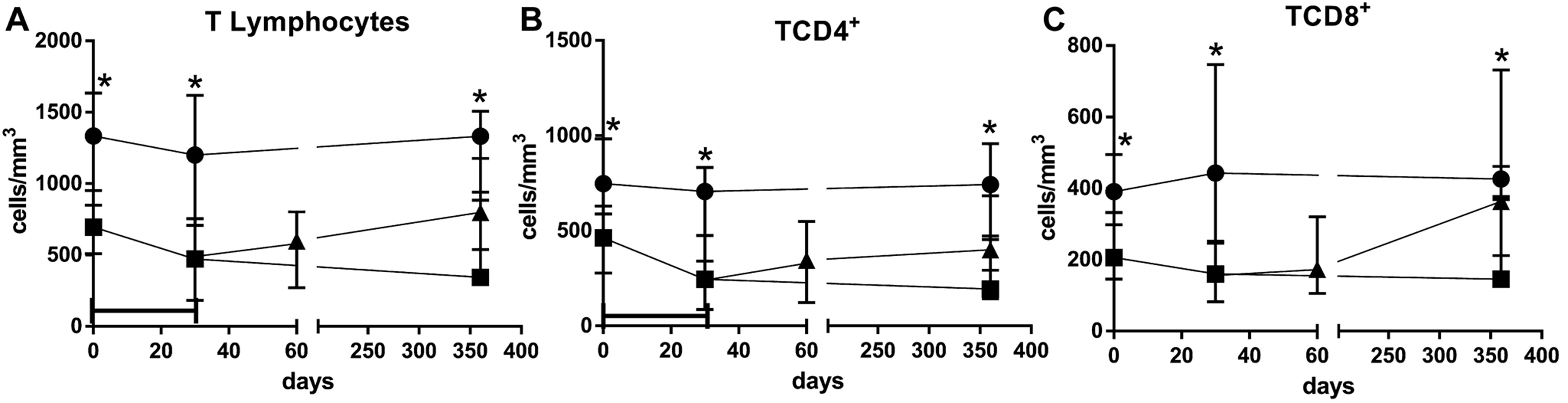

Supplement: Supplementary Figure 1 — T (A), TCD4+ (B) and T CD8+ (C) lymphocyte absolute counts over 365 days observation time for young standard immunosuppression (GROUP A1) (•), elderly standard immunosuppression (GROUP B1) (▪) and elderly everolimus conversion (GROUP B2) (▴) groups. * p<0.05 for comparison between GROUP A1 vs. GROUP B1 groups in a given time point. Horizontal bar p<0.05 for comparisons between 30 day and baseline cell counts of GROUP B1 group. [file Image1.jpeg]
